# Supplementary figures and images for: A mouse model of classical trigeminal neuralgia via intradural compression of the trigeminal nerve
Source: J Headache Pain. 2025 Dec 23;27(1):34. doi: 10.1186/s10194-025-02234-6 (PMC12860069; doi:10.1186/s10194-025-02234-6)

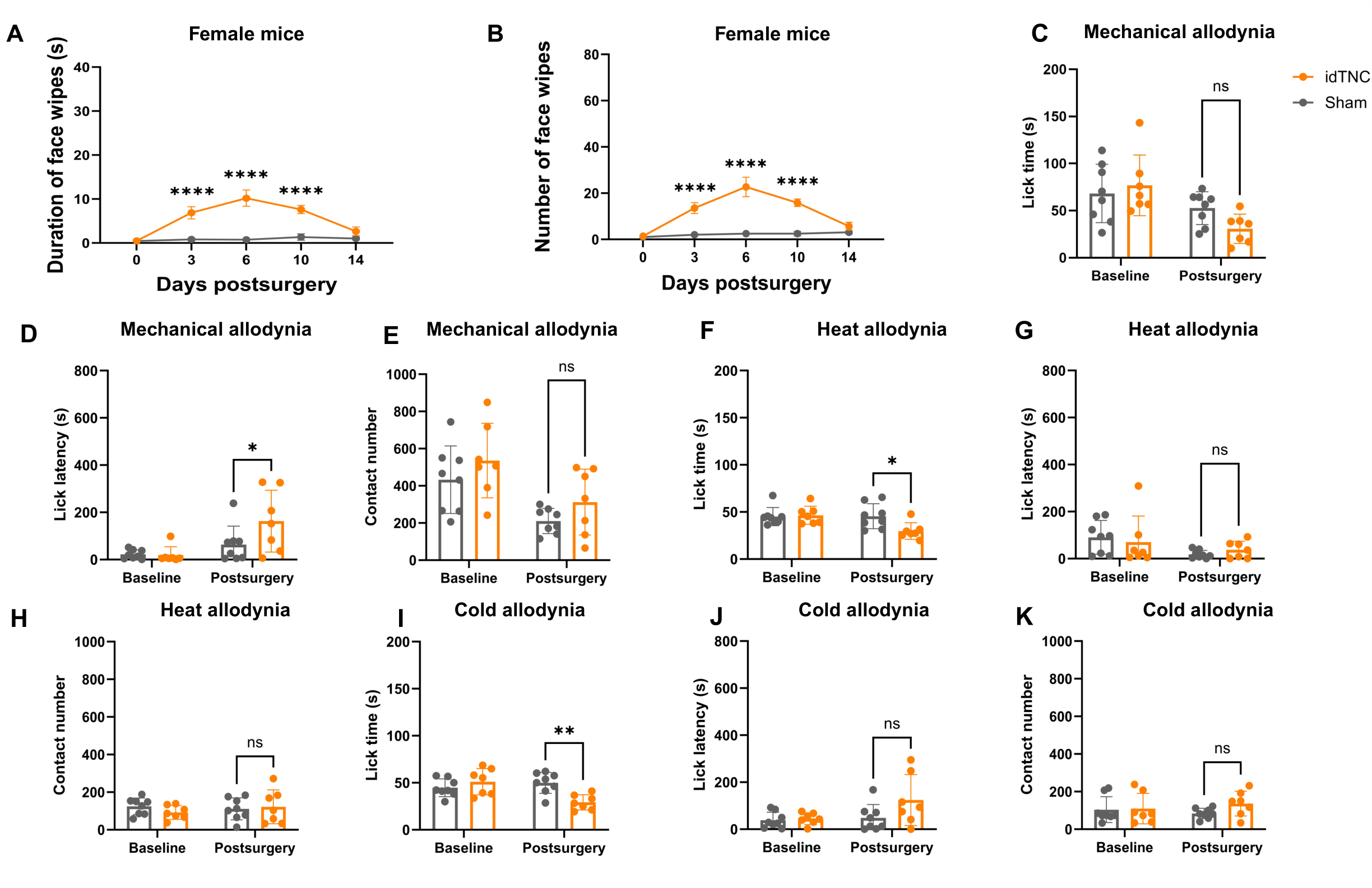

Supplement: Supplementary file 1 — Supplementary Material 1: Figure S1. The idTNC model results in spontaneous and evoked facial pain in female mice. (A, B) female mice that underwent idTNC surgery showed a higher duration (A) and number (B) of face-wiping episodes compared with sham mice on postoperative days (PODs) 3, 6, and 10. (C–K) Evoked pain assessment by OPAD. In the mechanical allodynia test, idTNC mice showed no significant difference in lick time (C) but a significant increase lick latency (D) compared with sham-operated mice. (E) The number of contacts was comparable between groups in the mechanical allodynia test. (F, G) idTNC mice showed significantly reduced lick time but no difference in lick latency in the heat allodynia test. (H) Contact numbers were similar between idTNC and sham-operated mice in the heat allodynia test. (I, J) idTNC mice exhibited significantly decreased lick time and a similar lick latency in the cold allodynia test. (K) Contact numbers were similar between idTNC and sham-operated mice in the cold allodynia test. (Female mice n = 7 for idTNC, n = 8 for sham surgery). Statistical analysis: two-way ANOVA with Sidak’s multiple comparisons test. *P < 0.05, **P < 0.01, ****P < 0.0001; ns, not significant. OPAD, orofacial pain assessment device. [file 10194_2025_2234_MOESM1_ESM.tiff]

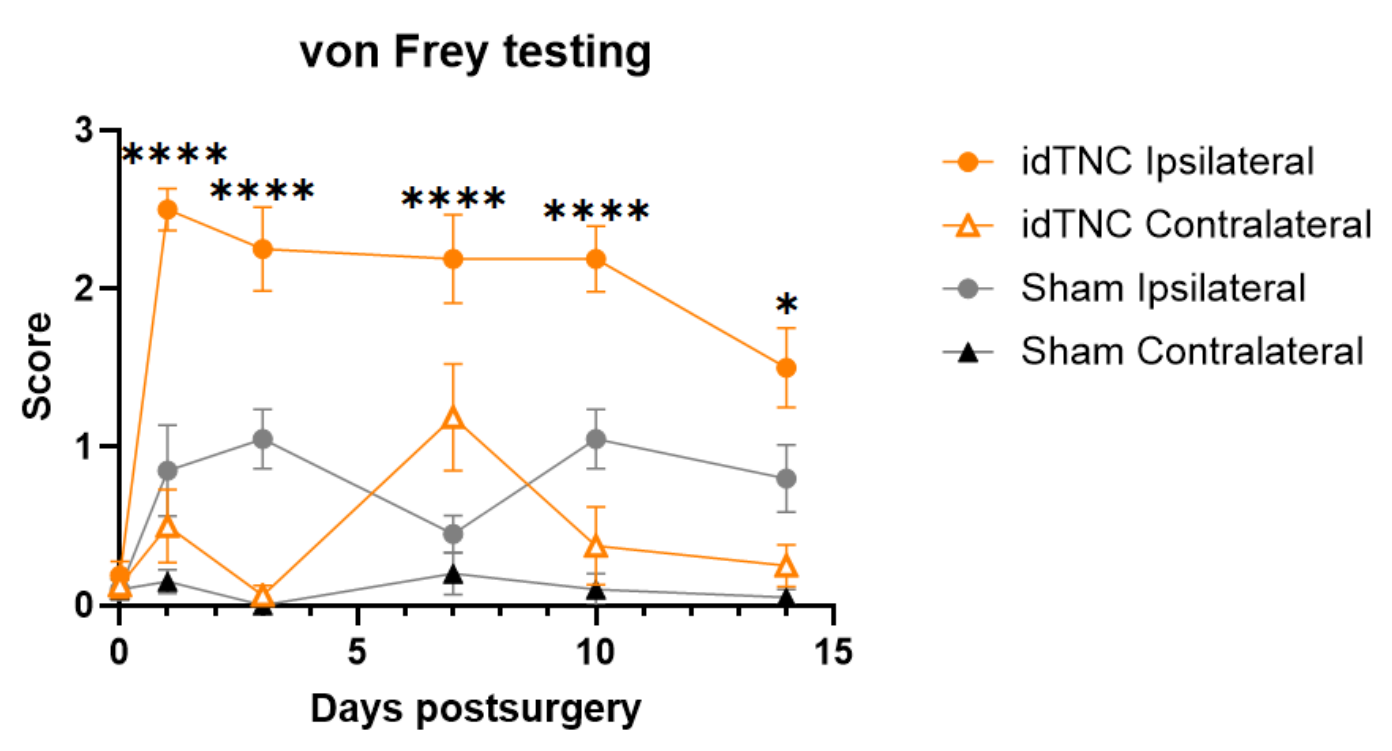

Supplement: Supplementary file 2 — Supplementary Material 2: Figure S2. Mechanical allodynia measured by orofacial von Frey test. On post operative days 1, 3, 7, 10 and 14 idTNC mice exhibited significantly higher mechanical sensitivity on the ipsilateral side compared with sham mice. Data are shown as mean values with ± SEM. (n = 8 for idTNC, n = 10 for sham surgery) Statistical analysis: two-way ANOVA with Tukey’s multiple comparisons test. *P < 0.05, ****P < 0.0001 [file 10194_2025_2234_MOESM2_ESM.tiff]
